# Decreased anterograde transport coupled with sustained retrograde transport contributes to reduced axonal mitochondrial density in tauopathy neurons

**DOI:** 10.3389/fnmol.2022.927195

**Published:** 2022-09-30

**Authors:** Anusruti Sabui, Mitali Biswas, Pramod Rajaram Somvanshi, Preethi Kandagiri, Madhavi Gorla, Fareed Mohammed, Prasad Tammineni

**Affiliations:** ^1^Department of Animal Biology, School of Life Sciences, University of Hyderabad, Hyderabad, India; ^2^Department of Systems and Computational Biology, University of Hyderabad, Hyderabad, India; ^3^Centre for Biotechnology, Institute of Science and Technology (IST), Jawaharlal Nehru Technological University Hyderabad, Hyderabad, India; ^4^Department of Biochemistry, School of Life Sciences, University of Hyderabad, Hyderabad, India

**Keywords:** axonal transport, kinesin, dynein, tau, Alzheimer’s disease (AD), mitochondria, P301L

## Abstract

Mitochondria are essential organelle required for neuronal homeostasis. Mitochondria supply ATP and buffer calcium at synaptic terminals. However, the complex structural geometry of neurons poses a unique challenge in transporting mitochondria to synaptic terminals. Kinesin motors supply mitochondria to the axonal compartments, while cytoplasmic dynein is required for retrograde transport. Despite the importance of presynaptic mitochondria, how and whether axonal mitochondrial transport and distribution are altered in tauopathy neurons remain poorly studied. In the current study, we have shown that anterograde transport of mitochondria is reduced in P301L neurons, while there is no change in the retrograde transport. Consistently, axonal mitochondrial abundance is reduced in P301L neurons. We further studied the possible role of two opposing motor proteins on mitochondrial transport and found that mitochondrial association of kinesin is decreased significantly in P301L cells. Interestingly, fitting our experimental data into mathematical equations suggested a possible rise in dynein activity to maintain retrograde flux in P301L cells. Our data indicate that decreased kinesin-mediated transport coupled with sustained retrograde transport might reduce axonal mitochondria in tauopathy neurons, thus contributing to the synaptic deficits in Alzheimer’s disease (AD) and other tauopathies.

## Introduction

Mitochondria play an essential role in neuronal health and survival. They supply the majority of ATP to support various processes in neurons, such as synaptic assembly, vesicle recycling, dendritic spine formation, and several forms of synaptic plasticity (Harris et al., 2012; Sun et al., 2013; Ashrafi and Ryan, 2017; Sheng, 2017). Besides, mitochondria play an important role in neuronal calcium signaling (Berridge, 1998; Bezprozvanny, 2009) and depletion of presynaptic mitochondria abrogates neurotransmission (Verstreken et al., 2005; MacAskill and Kittler, 2010; Sheng, 2017). Thus, proper distribution of mitochondria to synaptic compartments is essential for efficient neurotransmission. Accumulating evidence suggests that mitochondrial dysfunction is an early event in disease pathogenesis and contributes to synaptic deficits in AD and other neurological disorders (Lin and Beal, 2006; Wang et al., 2014; Swerdlow, 2018).

Because of their complex structure and the somatodendritic localization of biosynthetic machinery (Morfini et al., 2001; Pigino and Ishikawa, 2012), neurons rely on microtubule-based transport mechanisms to efficiently distribute cargo to the distal part of the axons. Mitochondrial transport is mediated by two opposing motor proteins, kinesin and cytoplasmic dynein. Kinesins regulate the anterograde movement of mitochondria away from the cell body. In contrast, cytoplasmic dynein drives mitochondrial transport towards the soma (Sheng and Cai, 2012; Moore and Holzbaur, 2018). In addition, neurons employ various adaptor proteins for spatio-temporal regulation of mitochondrial transport (Stowers et al., 2002; Kang et al., 2008; Reis et al., 2009; Zinsmaier et al., 2009; Misgeld and Schwarz, 2017). Defects in axonal transport are associated with many neurodegenerative disorders, including Alzheimer’s disease (AD) (Stokin and Goldstein, 2006; De Vos et al., 2008; Ari et al., 2014). Further, mutations in motor domains, impaired motor-cargo binding, or motor-microtubule interaction contribute to neuronal dysfunction (Puls et al., 2003; Braunstein et al., 2010; Brady and Morfini, 2017). Thus, investigating the pathophysiological mechanisms of mitochondrial transport might improve our current understanding of disease pathogenesis.

Tau is a microtubule-associated protein (MAP) abundantly present in axons and it undergoes phosphorylation at many sites (Goedert et al., 1993; Watanabe et al., 1993; Maurage et al., 2003). Hyperphosphorylated Tau forms neurofibrillary tangles in AD and other tauopathies (Wang and Mandelkow, 2016). Notably, the number of neurofibrillary tangles correlated well with cognitive deficits in AD (Giannakopoulos et al., 2003), suggesting the importance of tau phosphorylation in AD pathogenesis. Interestingly, several studies have shown that aberrant accumulation of phospho-tau is associated with mitochondrial defects and neuronal dysfunction in AD. For example, N-terminal truncated tau forms are localized to mitochondria (Atlante et al., 2008; Amadoro et al., 2011) and impair oxidative phosphorylation. Phosphorylated Tau interacts with VDAC1 (Manczak and Reddy, 2012) and parkin to impair mitochondrial quality control (Cummins et al., 2019). It has been shown that tau overexpression impairs mitochondrial transport in different cell types (Stoothoff et al., 2009; Vossel et al., 2010; Shahpasand et al., 2012). Though it is becoming clear that overexpression of wild-type, truncated, or phospho-tau inhibits mitochondrial transport (Alonso et al., 1994; Stamer et al., 2002; Gendron and Petrucelli, 2009; Kopeikina et al., 2011; Schulz et al., 2012; Shahpasand et al., 2012; Rodriguez-Martin et al., 2016; Quintanilla et al., 2020), molecular mechanisms behind these defects are not so clear.

In the current study, we have investigated the mechanisms underpinning the impaired mitochondrial transport in tauopathy neurons. Using primary cortical neurons and heterologous COS7 cells, our data indicate that P301L tau inhibits kinesin recruitment to mitochondria, thus reducing anterograde mitochondria movement towards the axon terminals. Further, our mathematical modeling suggests that changes in both antero- and retrograde transport lead to the overall decline in a net flux of mitochondrial transport, thus contributing to the reduced axonal mitochondrial density in tauopathy neurons.

## Materials and methods

### Materials

The WT construct encoding human MAPT (0N4R) was subcloned using gene-specific primers and P301L mutation was generated using a site-directed mutagenesis approach. MitoBFP, DsRed-Mito, and mCherry-Parkin plasmids are a kind gift from Prof. Naresh Sepuri at the University of Hyderabad. All the chemicals were obtained from the sigma unless otherwise mentioned. Antibodies were purchased from different sources: AT8 (#MN1020, Thermo Fisher Scientific, Waltham, MA, United States), GFP (JL-8, Takara Bio Clontech, Mountain view, CA, United States), LC3 (#L7543, Sigma, St. Louis, MO, United States), Actin (#A4700, Sigma, St. Louis, MO, United States), DIC (#MAB1618, Merck, NJ, USA), KIF5B (#ab9097, Abcam, United Kingdom), KIF1C (#20790-1-AP, Proteintech, Planegg-Martinsried, Germany), Tubulin (#T5293, Sigma, St. Louis, MO, United States).

### Cell culture and DNA transfection

COS7 cells were used to over-express wild-type and P301L tau. Cell lines used were tested and authenticated for contamination. COS7 cells are cultured in a DMEM medium with glutamine and 10% FBS and Pen-Strep mix. At 60% confluence, cells were transfected with Tau expression vectors using Lipofectamine 2000, according to the manufacturer’s instructions.

### Primary neuron cultures

Primary neurons were isolated from the cortical hemispheres of E16-18 mouse embryos as described previously (Feng et al., 2017; Tammineni et al., 2017b). Cortices were dissected from the embryos and subjected to papain digestion and dissociated cultures were plated on coverslips coated with poly-ornithine and fibronectin. Dissociated cultures were grown in neurobasal plating media (5% FBS, 1% Glutamax and 1X B27) overnight with L-glutamine supplement. From DIV 2, they were maintained in condition media with a one-third feed change every 2–3 days (1X B27 and Neurobasal). Transfections were performed using Lipofectamine 2000 between DIV4 to DIV5.

### Immunoblotting

For western blot analysis, COS7 cells expressing GFP fusion proteins were lysed in a lysis buffer (150 mM NaCl, 1% sodium deoxycholic acid, 1% Triton X-100, 0.1% SDS, 0.25 mM EDTA and 50 mM Tris–HCl, pH 7.2) with the protease and phosphatase inhibitor cocktail. Protein concentrations of the lysates were measured using the BCA reagent. An equal amount of lysates were resolved on SDS-PAGE and then transferred to a nitrocellulose membrane before probing them with antibodies of interest. The blots were developed using enhanced chemiluminescence reagents using Bio-Rad’s Chemi-doc imaging system.

### Isolation of mitochondria

Mitochondria were isolated from COS7 cells using the protocol described (Mohammed et al., 2020). Briefly, confluent COS7 cells were suspended in homogenization buffer (20 mM HEPES, pH 7.5, 1.5 mM MgCl2, 1 mM EDTA, 1 mM EGTA, 210 mM Sucrose and 70 mM Mannitol) and subjected to homogenization using Labgen125 homogenizer followed by 15-strokes of dounce homogenization. The homogenates were subjected to differential centrifugation at 800 × *g* for 10 min to remove the nucleus and debris. Further, supernatants were centrifuged at 10,000 × g for 10 min at 4°C to isolate mitochondria enriched fraction. Mitochondria enriched fraction was washed two times with homogenization buffer and finally suspended in resuspension buffer (250 mM Sucrose, 5 mM Magnesium acetate, and 10 mM HEPES-KOH pH 7.4). Before running them on SDS-PAGE, post-mitochondrial supernatants were subjected to TCA precipitation to prepare cytosolic extracts.

### Time-lapse and immunofluorescence imaging

Mouse cortical primary neurons were transfected with GFP or GFP fusion tau constructs and DsRed Mito for time-lapse imaging. Mitochondrial transport was monitored in the axons after carefully selecting them based on the morphology criteria described earlier (Feng et al., 2017; Tammineni et al., 2017a,b). The fasciculate axon or axon with many crossings was excluded from the quantification. Cells were visualized under the Leica SP8 microscope with a 63X oil immersion lens (1.4NA) to capture the transport dynamics using 488 nm excitation for GFP and 559 nm for DsRed Mito. A total of 100 frames were captured with an interval of 5 s. The stacks of the images were used to generate kymographs or QuickTime movies using NIH Image J software. To generate kymographs, we straightened curved axons and Z-projected re-sliced time-lapse images. The *Y*-axis of the kymograph represents recording time, while the *X*-axis represents the length of the axon imaged. Displacement over 5 μm was considered movement, while no change until the end of the recording was deemed to be stationary across all the frames. Percentage of mitochondria movement in anterograde and retrograde transport was measured for each axon by considering the number of mitochondria moving in either direction or stationary over the total mitochondria present and measurements were represented as SEM and statistical analysis was performed as described in the section “Methods.”

For confocal imaging, cells transfected with GFP Tau, MitoBFP, and mCherry Parkin were fixed with 4% paraformaldehyde after the treatments and images were acquired using Leica SP8 confocal microscope. Since Parkin is localized to mitochondria upon damage, we quantified Parkin localization to mitochondria. Cells were segregated based on the distribution of Parkin from being diffused to puncta and represented as SEM and statistical analysis was performed as described.

### Mathematical modeling of mitochondrial transport

We resort to a simple mathematical model to better understand the mechanism underlying the reduced axonal mitochondrial transport in tauopathy neurons. We developed a mathematical model to quantify the transport kinetics of mitochondria in P301L neurons to understand the effect of disease-associated phosphorylated tau on mitochondrial transport in neurons. We developed a mathematical model to quantify the transport kinetics of mitochondria in P301L neurons. The mitochondrial transport in neurons was modeled by assuming the passive facilitated transport framework. The forward transport (soma to axon) by kinesin motors and the retrograde transport (axon to soma) by dynein motors were considered for transport mechanisms in the model. The schematic of the mitochondrial transport used for modeling is shown in Figure 5A.

**FIGURE 1:**
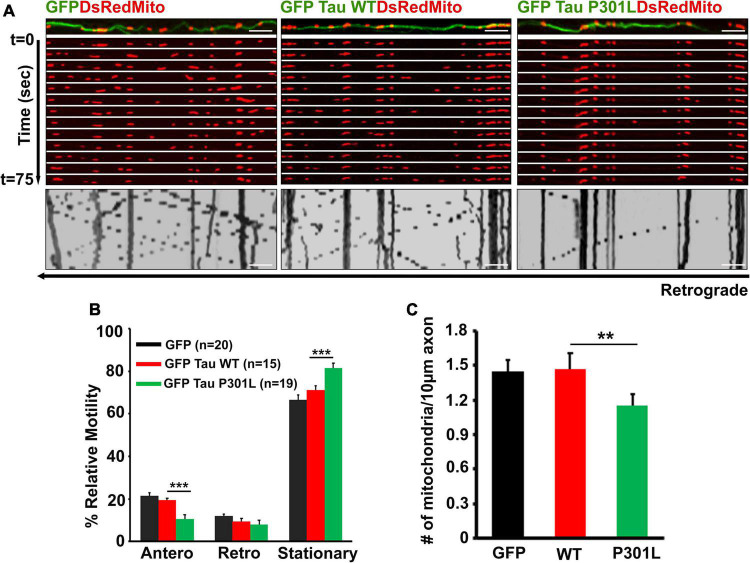
Overexpression of P301L reduce anterograde mitochondrial transport in mouse primary cortical neurons: **(A)** Representative time-lapse images and kymographs of GFP, GFP-Tau WT, and GFP-P301L expressing neurons showing the selective reduction of anterograde mitochondrial transport in P301L expressing neurons, but retrograde transport remains unaltered. Kymographs provide a two-dimensional representation of time-lapse imaging. In kymograph, stationary mitochondria are represented by vertical lines, while diagonal lines represent motile populations. Diagonal lines moving towards the left represent retrograde movement, whereas lines moving towards the right represent anterograde transport. **(B)** Mitochondrial motility in each direction was quantified and represented as % relative motility. **(C)** Quantitative analysis showing the reduction of mitochondria density in axons of P301L neurons. The number of mitochondria along the axons was counted and represented as the number of mitochondria per 10 μm axon. Data were obtained from three independent experiments. Number of cells used in the study are: GFP (*n* = 20 neurons; GFP Tau WT (*n* = 15); GFP P301L Tau (*n* = 19); (n) from three independent experiments. Scale bar = 5 μm. Error bars represent SEM. ***P < 0.001, **P < 0.01.

**FIGURE 2:**
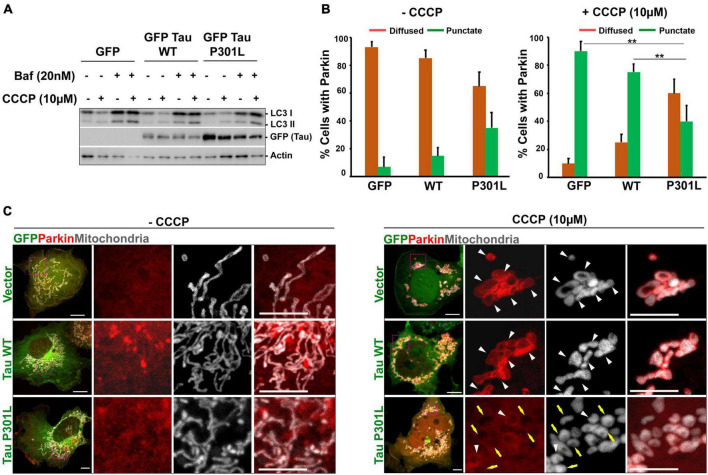
P301L inhibits parkin translocation to mitochondria in COS7 Cells: **(A)** Representative immunoblot to show that autophagy flux is not altered in cells expressing either wild-type or P301L tau. Cells were treated with CCCP (10 μM) to induce mitophagy. Bafilomycin (20 nM) was used to inhibit the fusion of autophagosomes with lysosomes. **(B)** Quantitative analysis representing the number of cells showing mitochondrial parkin (punctate) and cytosolic parkin (diffused) with and without CCCP treatment. Number of cells taken for quantification from three independent experiments (GFP: *n* = 29; GFP Tau WT: *n* = 30; GFP Tau P301L: *n* = 35). Multiple images were acquired from the random fields to measure the parkin translocation. **(C)** Representative confocal images showing the redistribution of parkin upon mitochondrial damage by CCCP. Cells co-expressing mCherry Parkin along with GFP, GFP Tau WT, and GFP Tau P301L were treated with 10 μM CCCP for 4 h. MitoBFP was used to visualize mitochondria. White triangle indicate the mitochondria with parkin, while yellow arrow indicates the defective parkin recruitment to mitochondria. Scale bar = 10 μm. Error bars represent SEM. **P < 0.01.

**FIGURE 3:**
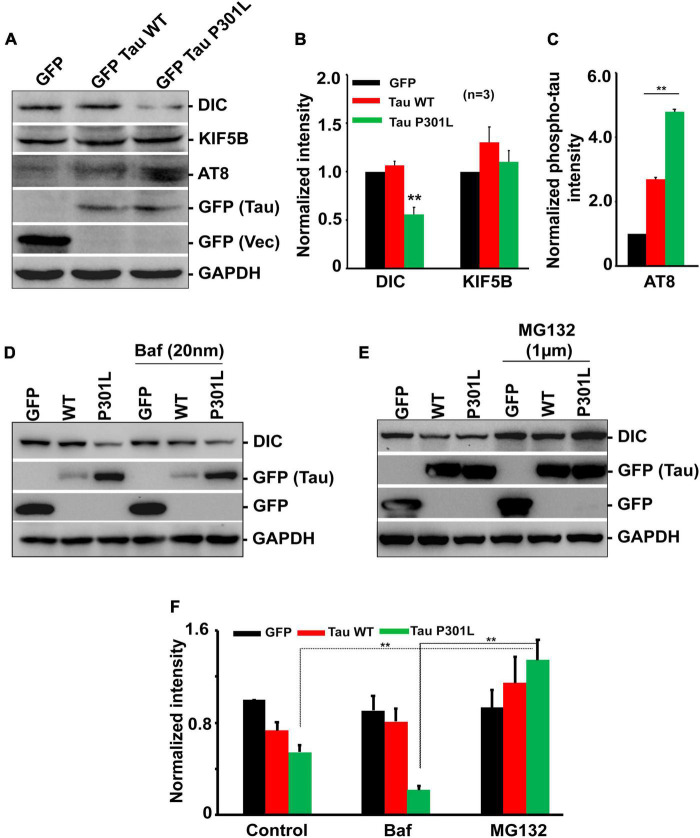
Overexpression of P301L reduced cytoplasmic dynein but did not affect kinesin protein levels. **(A)** Representative immunoblot and **(B,C)** Quantitative analysis showing the expression of dynein (DIC) and kinesin (KIF5B) protein levels in cells expressing GFP, GFP Tau WT, and GFP Tau P301L. Levels of dynein are reduced in cells expressing P301L tau. Further cells were treated with **(D)** Bafilomycin (20 nM) to inhibit autophagy or **(E)** proteasomal inhibitor, MG132 (1 μM), to examine the effect of two cellular degradation pathways on dynein protein turnover. MG132 restored dynein levels in GFP Tau P301L cells, indicating proteasomal-dependent degradation. **(F)** Quantitative analysis showing the expression of dynein during Bafilomycin (20 nM) and MG132 treatment. Data were obtained from three independent experiments, and error bars represent SEM. **P < 0.01.

**FIGURE 4:**
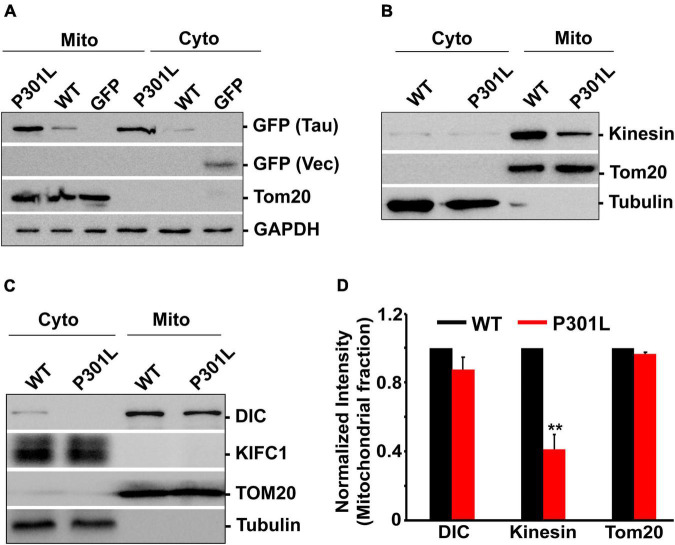
P301L inhibits kinesin recruitment to mitochondria: **(A)** Representative immunoblot showing the association of wild-type and mutant form tau with mitochondria. **(B,C)** Representative immunoblots showing the mitochondrial abundance of dynein and kinesin motors in cells expressing GFP Tau WT and GFP Tau P301L. Tom20, an outer mitochondrial membrane protein, was used as a mitochondrial marker. GAPDH or tubulin was used as cytosolic markers. KIFC1 was used as a negative control. **(D)** Quantitative analysis showing the mitochondrial association of kinesin and dynein in wild-type and P301L cells. Data were obtained from three independent experiments, and error bars represent SEM. **P < 0.01.

**FIGURE 5:**
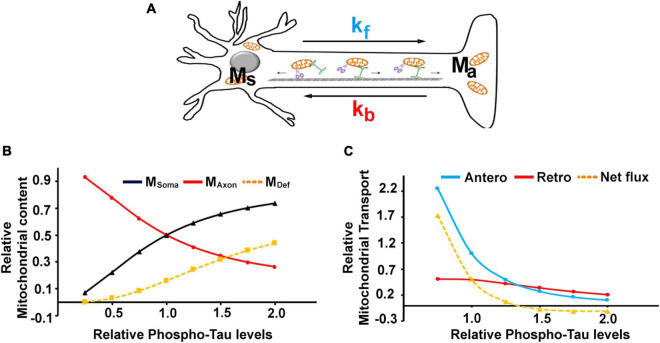
Mathematical modeling of mitochondrial transport in P301L neurons: **(A)** Schematic representation of parameters used to model the mitochondrial transport in neurons. M_soma_ and M_axon_ denote mitochondrial mass in the soma and axon compartments and M_Def_ denotes defective mitochondria. *k*_*f*_ and *k*_*b*_ represent kinesin and dynein mediated flux, respectively. Experimental datasets can be fitted into simple equations to simulate the effect of phospho-tau on **(B)** mitochondrial content and **(C)** transport parameters. The model was simulated for varying levels of Tau phosphorylation between 0.5 and 2-fold, where the level of tau phosphorylation in controls was considered 1 and 1.5 for the disease phenotype, as P301L displayed exhibited a 50% rise in phosphorylation.

The following equations model the transport process:

Transport scheme:

$$M_{s}\,\overset{K,k\,f}{\underset{D,k\,b}{⇄}}M_{A}$$

Where *M*_*s*_ and *M*_*A*_ are mitochondrial density/numbers in the soma (S) and axon (A). K and D denote kinesin and dynein levels, respectively. *k*_*f*_ and *k*_*b*_ are the rates of kinesin- and dynein-driven transport, respectively.

We assume that the total (*T*) mitochondrial mass (*M*_*T*_) is a sum of somal and axonal mitochondrial concentrations, as follows


(1) $$M_{T}=M_{A}+M_{s}$$

Assuming the rate of mitochondrial biogenesis and turnover is equal under the equilibrium conditions, we can write the mass balance equation for axonal mitochondrial concentration as:

Rate of change of axonal mitochondrial fraction = Kinesin mediated forward transport -Dynein mediated reverse transport


(2) $$\frac{d\,M_{A}}{d\,t}\;k_{f}*K*M_{s}-k_{b}*D*M_{A}$$

Where, *Kinesin mediated forward transport* =


(3) $$k_{f}*K*M_{s}$$

and *Dynein mediated reverse transport* =


(4) $$k_{b}*D*M_{A}$$

To quantify the effect of model parameters on mitochondrial transport, we solve equation (2) to obtain the steady-state solution for axonal mitochondrial concentration.

Substituting, $\frac{d\,M_{A}}{d\,t}=0$, we get

$$k_{b}*D*M_{A}=k_{f}*K*M_{s}$$

(5) $$M_{A}=\frac{k_{f}}{k_{b}}*\frac{K}{D}*M_{s}$$

Rearranging the equation (5), we get,


(6) $$\frac{M_{A}}{M_{s}}=\frac{k_{f}}{k_{b}}*\frac{K}{D}$$

Substituting Equation (1) in Equation (6) we get,


(7) $$\frac{M_{A}}{M_{T}-M_{A}}=\frac{k_{f}}{k_{b}}*\frac{K}{D}$$

(8) $$\frac{M_{A}}{M_{T}-M_{A}}=v*\frac{K}{D}$$

Equation (8) represents the fractional concentration of axonal mitochondria and the ratio

$v=\frac{k_{f}}{k_{b}}$represents the equilibrium constant for the transport process.

Further simplifying Equation (8) yields a relation between axonal mitochondrial concentrations to total mitochondrial concentration that follows hyperbolic (Michalis-Menten) kinetics,

$$M_{A}=\frac{v*K}{D+v*K}\,M_{T}$$

That can be written as


(9) $$M_{A}=\frac{1}{1+\left(\frac{D}{v*K}\right)}\,M_{T}$$

The above equation was further used to incorporate the effect of tau phosphorylation, for which the parameters were deduced based on the experimental observations and determine the steady-state values of the mitochondrial abundance in soma, axon, and anterograde and retrograde transport rates.

### Statistical analysis

All the data in the current study are presented as mean and standard error of the mean (SEM). Statistical analysis was performed using GraphPad Prism and statistical significance was calculated using paired *t*-test (for two group comparison) and two-way ANOVA-test with Fisher’s *post hoc* comparison was used for multiple groups. *P*-values ≤ 0.05 were considered significant and categorized as *P ≤ 0.05, **P ≤ 0.01, and ***P ≤ 0.001.

## Results

### Tau P301L inhibits mitochondrial transport in an anterograde direction but does not affect retrograde transport

Tau is a MAP primarily enriched in the axonal compartment of the neurons. It undergoes phosphorylation at multiple sites during the development and disease (Brion et al., 1993; Goedert et al., 1993; Yu et al., 2009). Accumulating evidence suggests that various tau forms inhibit mitochondrial transport, thus altering mitochondrial distribution. However, the molecular mechanisms underlying the transport defects are not so clear. We examined whether and how disease-associated tau mutation, P301L, affects mitochondrial transport based on these results. Tau P301L transgenic mice display progressive neuronal tauopathy by 6–7 months and develop motor impairments by 9–10 months (Terwel et al., 2005). Apart from neuropathology and cognitive deficits, it was shown that neurons from P301L mice display tau aggregates and mislocalization to dendrites. In addition, compared to other conformations of tau, the P301L mutant interacts less with microtubules; thus, it serves as an excellent model to study the effect of tau on organelle transport and its regulation (Terwel et al., 2005). To monitor the mitochondrial transport, we over-expressed DsRed-Mito in neurons expressing both wild-type and P301L tau. Mitochondrial movement is followed by time-lapse imaging (Supplementary Movie 1). As shown in Figures 1A,B, overexpression of wild-type tau did not affect either anterograde or retrograde transport of mitochondria. This is consistent with our previous work that control and wild-type tau expressing neurons display similar transport kinetics (Jeong et al., 2022). However, P301L tau selectively reduced anterograde mitochondrial transport but had minimal impact on retrograde transport in the same axon, ruling out the possibility of microtubule destabilization (Figure 1B). These data revealed that Tau P301L selectively inhibits anterograde transport of mitochondria while not affecting retrograde transport.

Next, we asked whether reduced mitochondrial transport impacts axonal mitochondrial density. Consistent with transport defects, overexpression of P301L significantly reduced the mitochondria number along the axons (Figure 1C). However, neurons expressing wild-type tau and GFP have the same mitochondrial density along the axons. Our data suggest that altered axonal mitochondrial transport might reduce mitochondrial density in the P301L neurons.

### Overexpression of P301L impairs Parkin translocation to mitochondria in P301L cells

To investigate the molecular mechanisms underlying reduced mitochondrial transport, we have generated a heterologous tauopathy model by overexpressing P301L in COS7 cells. COS cells provide an excellent model for understanding signaling aspects of tau-mediated transport, as tau is not present in COS cells (Chapman et al., 2019), ruling out the possible confounding effects of tau on microtubules. Cells expressing P301L show increased phosphorylation compared to either wild-type or GFP expressing cells (Figures 3A,C). This is similar to the previous work, as sites within the AT8 antibody epitope are phosphorylated in normal brain tissues but increased in tauopathies (Goedert, 1993; Watanabe et al., 1993; Maurage et al., 2003).

Mitochondrial dysfunction is reported in many tauopathy models and P301L inhibits mitophagy by inhibiting parkin translocation to mitochondria. CCCP was used to depolarize the mitochondria, as it acts as an uncoupling agent, dissipates mitochondrial membrane potential across the mitochondrial membrane, and thus promotes parkin recruitment (Zhu et al., 2014). To assess whether P301L inhibits Parkin translocation in COS7 cells, we co-transfected the cells with mCherry parkin and mito-BFP. 36 h post-transfection, cells were treated with CCCP for 4 h and parkin translocation to mitochondria was monitored. As shown in Figure 2C, CCCP treatment-induced parkin recruitment to mitochondria in control cells, though no parkin translocation was observed in DMSO treated control cells. However, a significant number of P301L cells showed reduced parkin translocation compared to wild-type or control cells, suggesting that P301L inhibits Parkin translocation in COS7 cells (Figures 2B,C).

Since damaged mitochondria are cleared through autophagy pathways, we also tested whether P301L affects the autophagy flux. To inhibit the autophagy process, cells were treated with 20 nM Bafliomycin A, widely used to inhibit autophagosome-lysosome fusion and lysosomal acidification (Mauvezin and Neufeld, 2015). Interestingly, cells expressing P301L have normal autophagic flux, though they show reduced Parkin recruitment to mitochondria (Figure 2A). This data suggests that COS7 cells expressing P301L tau display compromised mitochondrial quality control, similar to other neuronal cells (Cummins et al., 2019).

### Overexpression of P301L reduced dynein levels but did not affect kinesin motor levels

Because of the complex structural geometry, neurons rely on mitochondrial transport to efficiently distribute mitochondria to different sub compartments. Mitochondrial movement between the sub-compartments is governed by the association of these organelles with kinesin and dynein motors. Kinesin motors move the mitochondria away from the cell body, while dynein mediates retrograde transport of the mitochondria. Altered expression or mutations in these motor proteins leads to defective mitochondrial transport, thus reducing the mitochondrial abundance in axons (Ari et al., 2014; Hares et al., 2017; Chaudhary et al., 2018).

Since P301L reduced mitochondrial transport in an anterograde direction, we sought to determine whether the reduced expression of motor proteins contributes to the altered mitochondrial transport. We monitored the levels of kinesin, an anterograde motor protein, and dynein, a retrograde motor protein. Overexpression of GFP or wild-type tau did not affect either Kinesin or Dynein levels. However, P301L specifically reduced dynein levels, though levels of kinesin remain the same (Figures 3A,B). Further, reduced dynein levels are restored upon treatment with a proteasomal inhibitor, MG132 (Figures 3E,F), suggesting the role of the proteasomal pathway in dynein turnover in P301L cells. However, the bafilomycin (20 nM) treatment did not rescue the reduced dynein levels in P301L cells (Figures 3D,F).

To further assess whether P301L affects the mitochondrial association of the motor proteins, we isolated mitochondrial fractions from the cells expressing wild-type and P301L cells. The purity of mitochondrial fractions was confirmed by abundance pf Tom20, an outer mitochondrial membrane protein. Tubulin was used as a cytosolic marker. As shown in Figure 4A, both wild-type and P301L proteins are associated with mitochondria, consistent with the previous report (Atlante et al., 2008; Amadoro et al., 2011). However, mitochondrial levels of kinesin were significantly reduced in P301L cells (Figures 4B,D). Surprisingly, dynein levels on mitochondria were moderately affected despite the reduced expression of dynein in P301L cells (Figures 4C,D). KIF1C, a motor protein involved in organelle and chromosomal movement, remains in the cytosol in wild-type and P301L expressing cells. Since Kinesin driven anterograde transport delivers mitochondria to the axons and dynein retracts mitochondria from the axon, our data suggest that reduced Kinesin recruitment to mitochondria leads to defective anterograde transport, while maintaining stable retrograde flux in P301L cells, which might contribute to the altered mitochondrial density along the P301L axons.

### Mathematical modeling of mitochondrial transport in P301L neurons

Mitochondrial transport along the axon is a dynamic process. Kinesin motors contribute to the forward flux, while dynein motors contribute to the reverse flux. Given the opposing nature of the motors, the distribution of mitochondria along the axons is a function of both forward and reverse flux. Since phosphorylation of tau in P301L cells was increased in our experimental dataset as compared to the wild-type, we used simple mathematical equations to characterize the features of mitochondrial transport in P301L cells as a function of phosphorylated tau. Our model is based on the following assumptions. First, we assumed the baseline model response corresponding to the control phenotype from GFP or Tau WT expressing cells. Second, the control’s model parameters were approximated to 1 (*k*_*f*_, *k*_*b*_, *K*, *D* = 1), as we always represented experimental data of P301L cells with respect to wild-type tau and control. Third, total mitochondrial mass (M_*T*_) is a sum of mitochondria fraction present in the soma compartment (M_*s*_) and axon compartment (Ma) and is equal to 1.

Based on these assumptions, we evaluated the model parameters for the effects of tau phosphorylation on transport rates and protein expression to validate our experimental data from P301L expressing neurons. Our experimental data shows that P301L tau significantly affects kinesin driven mitochondrial transport (*k_f*) and dynein expression (D), both of which are reduced by 50% compared to control or wild-type tau. Assuming that the effects of P301L are mainly due to an increase in phosphorylation of tau, we simulated the model for varying levels of Tau phosphorylation between 0.5 and 2. Since we represented the data from P301L neurons as fold change with respect to control (where 1 is considered as phosphorylation status in control and 1.5 for disease-associated tau phosphorylation) (Figures 5B,C), these values represent the extrapolation of our experimental observations. This value range allow us to have the empirical data corresponding to phosphorylated tau equal to 1 (control) and 1.5 (P301L) as the ratio would remain same for any basal value of tau when considered as fold change. Therefore, we used 0.5 and 2 as the mathematical inputs to the model to extrapolate the response at different values of tau phosphorylation that however, also satisfies the experimental observations for 1 and 1.5-fold tau phosphorylation on neuronal mitochondrial transport.

The model parameters for the effect of phosphorylated tau in P301L cells were determined in such a way that the observations from our experiments for control and P301L cells cases can be deduced using the model. Therefore, the experimental datasets for control and P301L cases correspond to the two data points on the curves generated using the model.

By fitting the experimental data points with the equations, we extrapolated the datasets to obtain model parameters as a function of the phosphorylation status of tau. As shown in Table 1, the model output was comparable to experimental observations.

**TABLE 1:** Significant differences observed in tauopathy neurons with respect to controls and the mathematical model validation.

Control/Wild-type tau vs. P301L tau	% Change obtained from experimental data	Fold change	% Change from mathematical modeling
Drop in anterograde transport (*k*_*f*_ × *K* × *M*_*s*_)	73	0.27	73
Drop in retrograde transport (*k*_*b*_ × *D* × *M*_*A*_)	30	0.70	32
Decrease in Axonal Mitochondria (*M*_*A*_)	33	0.67	31
Decrease in Dynein expression (*D*)	50	0.50	45
Decrease in Kinesin recruitment (*k*_*f*_)	50	0.50	48
Increase in defective Mitochondrial (*M*_*D*_)	95	1.95	95
Increase in dynein processivity (*k*_*b*_)	NA	1.80	80
Drop in net forward transport (*k*_*f*_ × *K* × *M*_*s*_)- (*k*_*b*_ × *D* × *M*_*A*_)	108	−0.08	114

The phenomenological equations derived to simulate the effect of P301L on model parameters were derived assuming Hill-type kinetics and are given by Equations 10–12, where tau (τ) indicates tau phosphorylation relative to control.

Effect of tau phosphorylation on kinesin activity/recruitment:


(10) $$\tau_{k_{f}}=\omega \,1*\left(\frac{1}{1+\tau^{n\,1}}\right),\text{where}\,\omega \,1=2\,\mathrm{and}\,n1=2.6.$$

Effect of Tau phosphorylation on dynein expression:


(11) $$\tau_{D}=\omega \,2*\left(\frac{1}{1+\tau^{n\,2}}\right),\text{where}\,\omega \,2=2\,\mathrm{and}\,n2=2.4.$$

Effect of Tau phosphorylation on dynein activity


(12) $$\tau_{k_{b}}=\omega \,3*\left(\frac{1}{1+{\left(\frac{2}{\tau }\right)}^{n\,3}}\right),\text{where}\,\omega \,3=5\,a\,n\,d\,n\,3=2.$$

The functions (τ_*Kf*_, τ_*D*_, and τ_*Kb*_) and values of ω and *n* were chosen such that the fold changes in kinesin activity, dynein expression and dynein activity were reproduced with respect to changes in tau phosphorylation.

We further observed that to rationalize the experimental observations in the disease phenotype, our model predicted an increase in the dynein activity in P301L cells (Table 1) by 1.8-fold due to the effect of Tau phosphorylation, a qualitative trend as reported earlier (Miller and Sheetz, 2004; Quintanilla et al., 2020). It is corroborated by *in vitro* reconstituted experiment suggesting that tau biases to dynein-mediated retrograde transport while inhibiting the kinesin-mediated anterograde transport. (Vershinin et al., 2007; Chaudhary et al., 2018).

By introducing these effects (Equations 10–12) in the model [Equation (7)], we get,


(13) $$\frac{M_{A}}{M_{T}-M_{A}}=\frac{k_{f}*\omega \,1*\left(\frac{1}{1+\tau^{n\,1}}\right)}{k_{b}*\omega \,3*\left(\frac{1}{1+{\left(\frac{2}{\tau }\right)}^{n\,3}}\right)}*\frac{K}{D*\omega \,2*\left(\frac{1}{1+\tau^{n\,2}}\right)}$$

(14) $$\frac{M_{A}}{M_{s}}=R*\left(\frac{1+\tau^{n\,2}}{1+\tau^{n\,1}}\right)*\left(1+{\left(\frac{2}{\tau }\right)}^{n\,3}\right)*\frac{K}{D}$$

Where *R* is the strength of an effect of tau phosphorylation on the transport process given by,

*R* = ω * *v*, ω $=\frac{\omega \,1}{\omega \,2*\omega \,3}$ and $v=\frac{k_{f}}{k_{b}}$

Since $n\,1\approx n\,2,\left(\frac{1+\tau^{n\,2}}{1+\tau^{n\,1}}\right)\approx 1$, we can approximate Equation (14) as


(15) $$\frac{M_{A}}{M_{s}}=R*\left(1+{\left(\frac{2}{\tau }\right)}^{n\,3}\right)*\frac{K}{D}$$

Therefore, the steady-state equation for axonal mitochondrial mass can be given by,


(16) $$M_{A}=\frac{M_{T}}{1+\left(\frac{D}{R*\left(1+{\left(\frac{2}{\tau }\right)}^{n}\right)*K}\right)}$$

where *R* = (1/ ω3) = 0.2, D = K = 1, n = n3 = 2


(17) $$M_{A}=\frac{M_{T}}{1+\left(P*\left(\frac{\tau^{n}}{\tau^{n}+2^{n}}\right)*\frac{D}{K}\right)}$$

Where *P* = 1/*R* = 5, and $P*\left(\frac{\tau^{n}}{\tau {+}^{n}2^{n}}\right)$ is Tau dependent net dynein processivity rate.

By introducing these effects into the model, we simulated the changes in mitochondrial abundance in axons and soma as a function of tau phosphorylation using equation (13). As shown in Figure 5B, mitochondrial mass was changed with increasing tau, while somal mitochondrial content was increased. It is consistent with the previous study that over-expression of P301L increased somal mitochondrial density (Quintanilla et al., 2020).

Since damaged mitochondria are not transported efficiently in the anterograde direction (Miller and Sheetz, 2004; Quintanilla et al., 2020), our model also predicted that the number of defective mitochondria appears to increase with the phosphorylation status of tau (Jeong et al., 2022). This is also similar to an increase in the faulty mitochondria in both axons and soma of P301L neurons (Jeong et al., 2022). The quantity of defective mitochondria was proportional to reduced kinesin activity and mitophagy induced by tau phosphorylation (Jeong et al., 2022). So, we considered mitophagy correlates with tauopathy as the function of τ_*kf*_ and estimated it as an inverse function of the effect of tau phosphorylation on kinesin activity.

Therefore, the *M*_*D*_ (M_*Def*_ in Figure 5B) concentrations were evaluated by taking the difference between the normal axonal mitochondria levels and the axonal mitochondrial levels that would be due to τ_*kf*_ (i.e., reduction in kinesin activity due to tau phosphorylation) is given by:


(18) $$M_{D}=M_{A\,b}-M_{A\,\tau }$$

Where, *M*_*Ab*_ is the axonal mitochondria levels at baseline tau phosphorylation and kinesin activity, and *M*_*Aτ*_ is the axonal mitochondrial levels on increasing tau phosphorylation and resultant decrease in kinesin activity. *M*_*Aτ*_ can be written by substituting Equation10 in Equation 9 as


(19) $$M_{A\,\tau }=\left(\frac{M_{T}}{1+\left(\frac{D}{v*\omega \,1*\left(\frac{1}{1+\tau^{n\,1}}\right)*K}\right)}\right)$$

From Equations 18, 19 the concentrations for defective mitochondria can be approximated as,


(20) $$M_{D}=M_{A\,b}-\left(\frac{M_{T}}{1+\left(\frac{D}{v*\omega \,1*\left(\frac{1}{1+\tau^{n\,1}}\right)*K}\right)}\right)$$

The steady-state mitochondrial transport rates were determined by Equations 3, 4 and plotted in Figure 5C. The effects of phosphorylated tau on anterograde transport appear to be more prominent, as the steepness of the anterograde curve is higher than retrograde transport (Figure 5C). It can also be noted that the net forward flux (Equation 3 – Equation 4) tends to be negative with increasing tau phosphorylation (for τ > 1.25). At the same time, there is a relative increase in the retrograde transport, despite the reduced expression of dynein protein. Together, our model suggests that altered mitochondrial distribution of mitochondria in P301L neurons is contributed by reduced anterograde transport and sustained retrograde transport.

## Discussion

Neurons are polarized cells with complex architecture. Since diffusion rates of ATP are limited, presynaptic mitochondria supply most of the energy, in the form of ATP, required for neurotransmission (MacAskill and Kittler, 2010; Sheng, 2017). Thus, maintaining the healthy mitochondria in the synaptic terminals is vital for neuronal function and plasticity (Ma et al., 2009; Sheng, 2014). Though mitochondrial dysfunction is associated with many neurological disorders, the pathophysiological mechanisms associated with mitochondrial transport are not completely understood. In this context, it has been reported that tau impairs mitochondrial transport (Alonso et al., 1994; Gendron and Petrucelli, 2009; Stoothoff et al., 2009; Vossel et al., 2010; Schulz et al., 2012; Shahpasand et al., 2012; Quintanilla et al., 2020), yet there is no unifying mechanism for how pathological forms of tau regulate mitochondrial transport. This is partly because different studies have used various forms of tau (Quintanilla et al., 2020) and diverse cell types (Stoothoff et al., 2009; Vossel et al., 2010). In addition, most of the studies focused on anterograde transport, as it supplies mitochondria to the axons and synaptic terminals. However, the role of cytoplasmic dynein in mitochondrial distribution is often overlooked. This is very important as dynein retracts mitochondria from the axon terminals, thus equally contributing to the mitochondrial distribution in axon and synaptic terminals.

In the current study, we have provided more evidence to substantiate the role of altered motor-cargo interactions as a contributing factor to mitochondrial transport defects in P301L neurons. Consistent with the previous reports (Jeong et al., 2022), overexpression of P301L in mouse primary cortical neurons reduced anterograde movement of mitochondria, while having minimal effect on retrograde transport. Our data revealed that such defects are caused by reduced recruitment of kinesin motors to mitochondria in P301L cells. The expression of kinesin protein, however, remains same in our heterologous COS7 system. Since aberrant activation of parkin, in P301L neurons, promotes the degradation of Miro1, a mitochondrial adaptor for kinesin (Jeong et al., 2022), it is possible that defects in kinesin recruitment are due to reduced availability of Miro1 in P301L cells. Alternatively, P301L tau may also sequester motor-adaptor complexes (Ittner et al., 2009) or affect the functional properties of these complexes by activating the upstream signaling pathways. Since tau expression in COS7 cells may interrupt the internal signaling, further studies are needed to gain more mechanistic insights using structurally complex neurons and validate our findings from heterologous COS7 cells.

Both kinesin and dynein motor proteins regulate the spatial and functional distribution of cargo in neurons. Yet many studies proposed that altered anterograde transport contribute to reduced axonal mitochondrial density in tauopathy neurons (Jeong et al., 2022). In the current study, we proposed that dynein mediated retrograde transport might play equally important role in regulating mitochondrial distribution in tauopathy neurons (Combs et al., 2019; Jeong et al., 2022). Further, we have shown that levels of dynein in the mitochondria fraction were moderately decreased despite a significant reduction in total dynein protein expression. Consistently, retrograde transport in P301L neurons remains the same. However, the physiological and functional relevance of reduced dynein protein expression coupled with sustained retrograde transport remains to be studied further. It has been shown that CDK5 regulates dynein force adaptation for mitochondria and lysosomes (Chapman et al., 2019) by stabilizing the dynein-NudEL-LIS1 complex to enhance dynein force production. Since CDK5 is also activated in AD (Liu et al., 2016) and phosphorylates tau (Kimura et al., 2014), it is likely that decreased dynein in P301L cells might be an early adaptation mechanism to fine-tune the transport from being too robust in the retrograde direction. This is particularly important in neurons having compromised anterograde transport to efficiently utilize the sparse mitochondria available in axons during the early onset of disease. Otherwise, dynein-mediated transport robustly retracts most of the mitochondria to the soma compartment. Since retrograde transport facilitates the removal of damaged mitochondria (Cai et al., 2012; Devireddy et al., 2015), it is also possible that neurons maintain sustained retrograde transport to remove the damaged mitochondria to maintain the axonal mitochondrial integrity during the early onset of the disease. Consistently, fitting our experimental observations into simple mathematical modeling suggests that reduced anterograde transport and stable retrograde transport contribute to decreased axonal mitochondrial mass in P301L neurons. Our model also predicted that somal mitochondrial mass is increased in P301L neurons, which is consistent with the previous reports (Quintanilla et al., 2020). Together, our data suggest that reduced anterograde transport and stable retrograde transport depletes axonal mitochondria in P301L cells, thus contributing to synaptic energy deficits. Our studies provide a basic frame work for understanding the motor-adaptor-cargo interactions in regulating the spatial and functional distribution of mitochondria in tauopathy. However, further studies are required to validate the dynamic interplay of these pathological mechanisms and how they contribute to synaptic deficits in AD and other tauopathies.

## Data availability statement

The raw data supporting the conclusions of this article will be made available by the authors, without undue reservation.

## Ethics statement

This animal study was reviewed and approved by Institutional Animal Ethical Committee (IAEC), University of Hyderabad (#UH/IAEC/PT/2019-I/12).

## Author contributions

AS, MB, PK, and FM: experimentation and data analysis. PS: mathematical modeling of mitochondrial transport and data analysis. MG: experimentation, data analysis, and writing. PT: conception, experimentation, analysis, and writing the manuscript. All authors contributed to the article and approved the submitted version.

## Supplementary material

Supplemetary Video 1 GFP DsRed Mito.

Supplemetary Video 2 GFP Tau P301L-DsRed.

Supplemetary Video 3 GFP Tau P301L-DsRed.
